# Supplementary material for: A versatile cryo-transfer system, connecting cryogenic focused ion beam sample preparation to atom probe microscopy
Source: PLoS One. 2021 Jan 19;16(1):e0245555. doi: 10.1371/journal.pone.0245555 (PMC7815152; doi:10.1371/journal.pone.0245555)
Supplement: S1 File — (DOCX) [file pone.0245555.s001.docx]

**A versatile cryo-transfer system, connecting cryogenic focused ion beam sample preparation to atom probe microscopy**

**Videos of important points in the transfer process**

Video S1The hand off between the FIB cryo shuttle on the FIBTD and the Cu-sheath of the ACTD

Video S2 The transfer of the double nipple from the ACTD into the LEAP

**Temperature measurements during transfer**

To confirm temperature and pressure during important steps of the transfer, when the sample is not actively cooled, a thermocouple and required fittings were attached to the FIB transfer device (FIBTD), FigS1, A and B. The thermocouple wire was attached directly to the double nipple and the temperature was measured while moving the FIB cryo shuttle from the cryo stage (set point: -120° C) and throughout the hand-off of the double nipple from the FIBTD to the actively cooled transfer device (ACTD). The hand-off from the ACTD to the custom LEAP puck is not investigated here, as it is very similar to handoff from the FIB puck to the atom probe transfer arm with even shorter transfer times (< 10 s), at better pressures (< 1x10^-7^ mbar), with both ends actively cooled to liquid nitrogen temperatures.

These measurements were carried out with thermocouples attached directly to the FIB cryo shuttle and double nipple, Fig S1, C and D. This is not possible during regular operation, since it requires the thermocouples to be fixed to the individual parts. Thus, a continuous measurement during the entire transfer is not possible. It demonstrates, however, the temperatures and cooling/heating rates to be expected during transfer. In normal operation, the temperature close to the sample is monitored (described in the main text) as well as pressure.


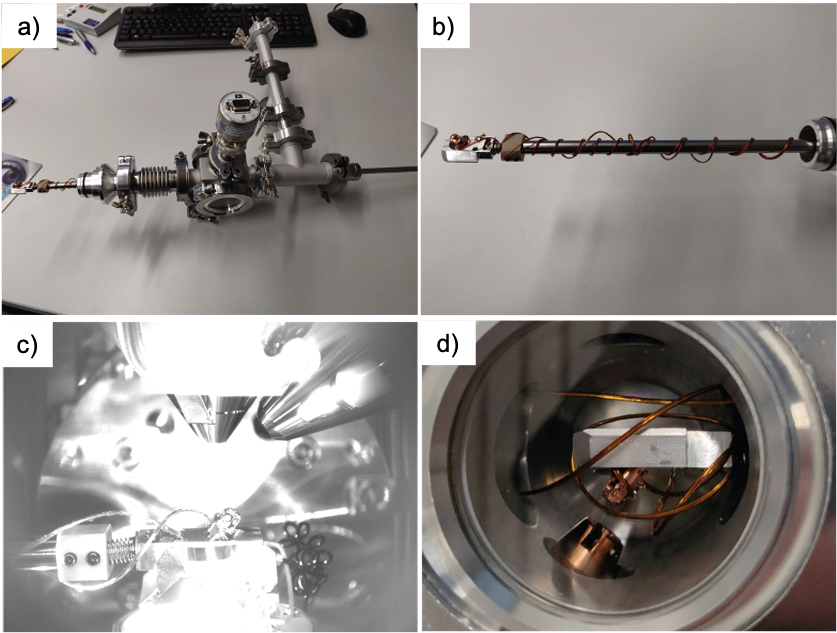


Fig S1, The experimental setup of the temperature measurements during transfers. a) new components connected to the FIBTD enabled temperature measurements. b) a thermocouple was attached to the FIB cryo shuttle, wrapping around the transfer rod to enable temperature to be measured when c) moving off of the cryo FIB stage. The thermocouple was also attached to the double-nipple in d) to measure temperature during hand-off to the ACTD.

*Pressure and temperature log during nipple handover*

In Fig S2, a nipple was detached from a FIB cryo-shuttle (cooled to -118° C) and attached to the ACTD. After cooling to T< -160° C, the double nipple was reattached to the FIB cryo-shuttle on the FIBTD, since it had a thermocouple wire attached to it. While the double nipple was attached to the ACTD, the temperature of the FIB cryo shuttle, which is not actively cooled, was measured. The FIB cryo shuttle was intentionally cooled to a higher temperature (using the cryo stage) to observe the cooling of the nipple in the ACTD.

This measurement also shows that the heating rate of the cooled FIB cryo shuttle during the hand-off is low (2.3 °C/min when starting at -118° C) at the low pressures provided by the FIB/SEM turbo pump (p<1 x 10^-5^ mbar), leaving plenty of time for the transfer. Typical transfer times in regular operation are ~1 min.


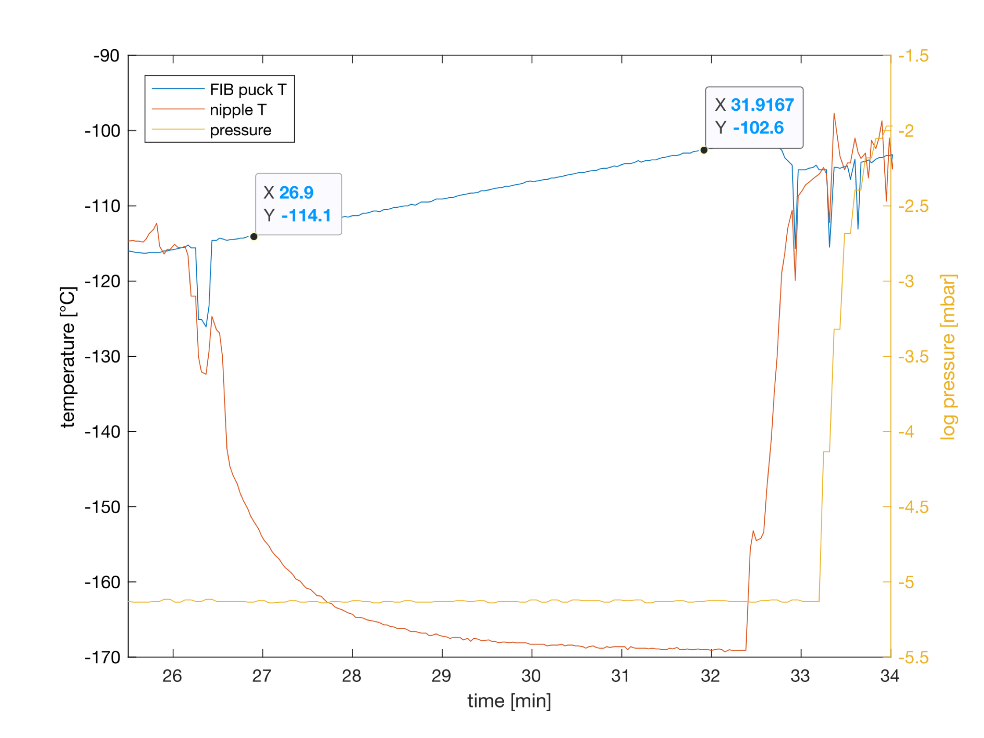


Fig S2, Temperature and pressure as a function of time when a cool sample is moved from the cryo-FIB stage, cooled further by the ACTD and then reattached to the FIBTD. Note: The vacuum gauge used only allows measurements down to 1 x 10^-5^ mbar

*The importance of pressure*

A double nipple in a FIB cryo shuttle was cooled to at least -90° C and the rate at which it warmed to -70° C was measured in different pressures, as shown in Table S1. It is important to note that these warming rates depend not only on pressure, but also the temperature of the FIB cryo shuttle relative to the FIBTD transfer rod (e.g. room temperature). These results clearly show how important it is to maintain low pressures to reduce sample warming (as well as frost formation).

Table S1 The heating rates of a double nipple cooled to -90° C at various pressures ranging from FIB/SEM operating pressure to atmosphere. This shows how important pressure is to slowing how quickly a sample warms up (not to mention frost formation).

| Pressure [mbar] | < 10^-5^  (2 x 10^-6^) | 7 x 10^-3^ | 3 x 10^-2^ | 6 x 10^2^ |
| --- | --- | --- | --- | --- |
| Heating rate [K/s]  -90°C to -70°C | 1.2 | 4.0 | 10.1 | 16.9 |

**Connection of APT transfer shuttle to sample preparation stations (other than FIB)**


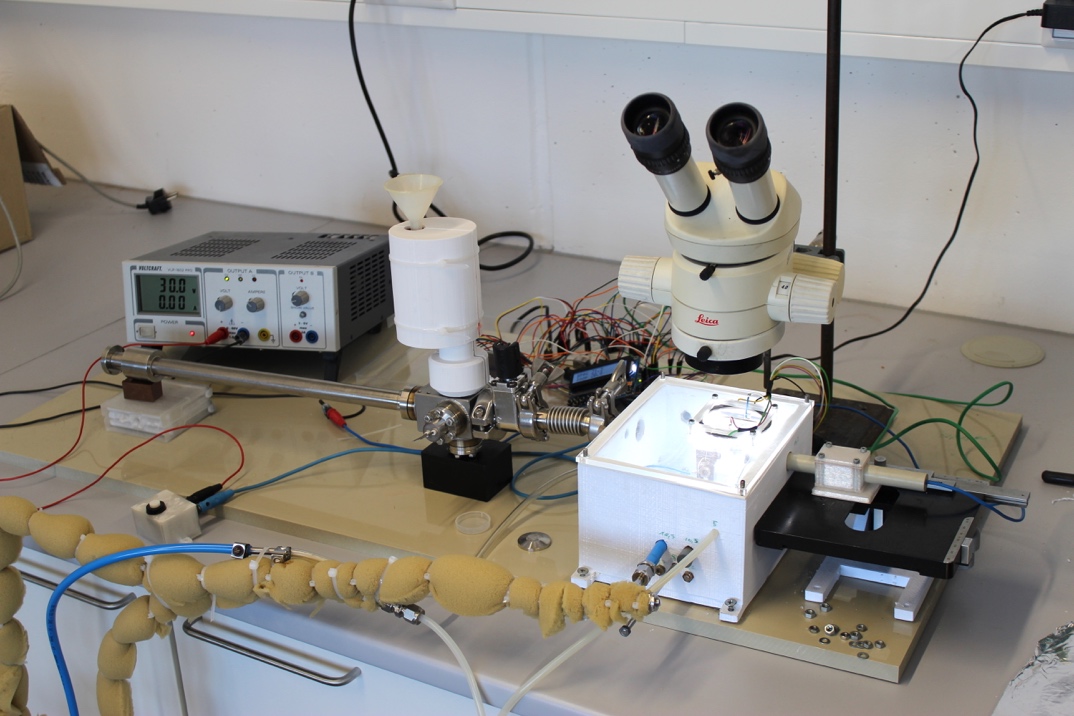


Fig S3, ACTD attached to cryo-electropolishing station


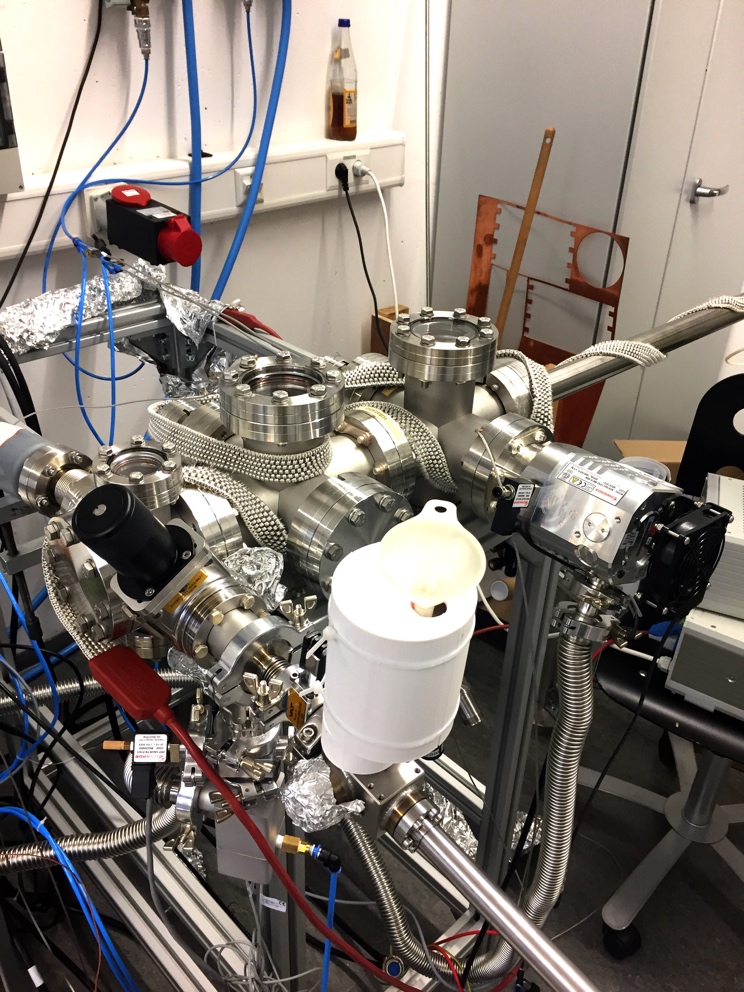


Fig S4, ACTD attached to PVD / ion etching unit

**Mass spectrum of the resulting APT data**


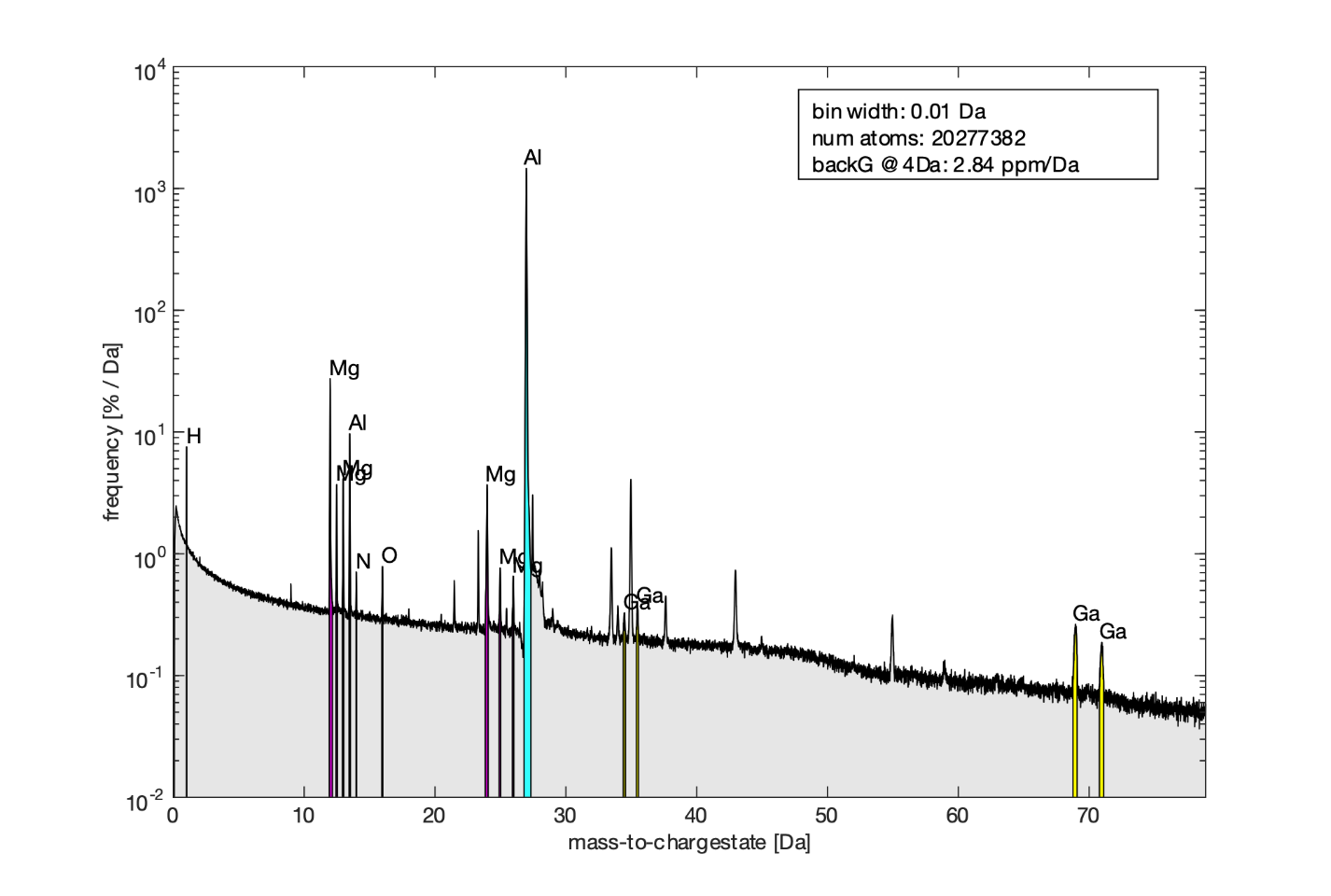


Fig S5, Overview of the mass spectrum of the atom probe data set.


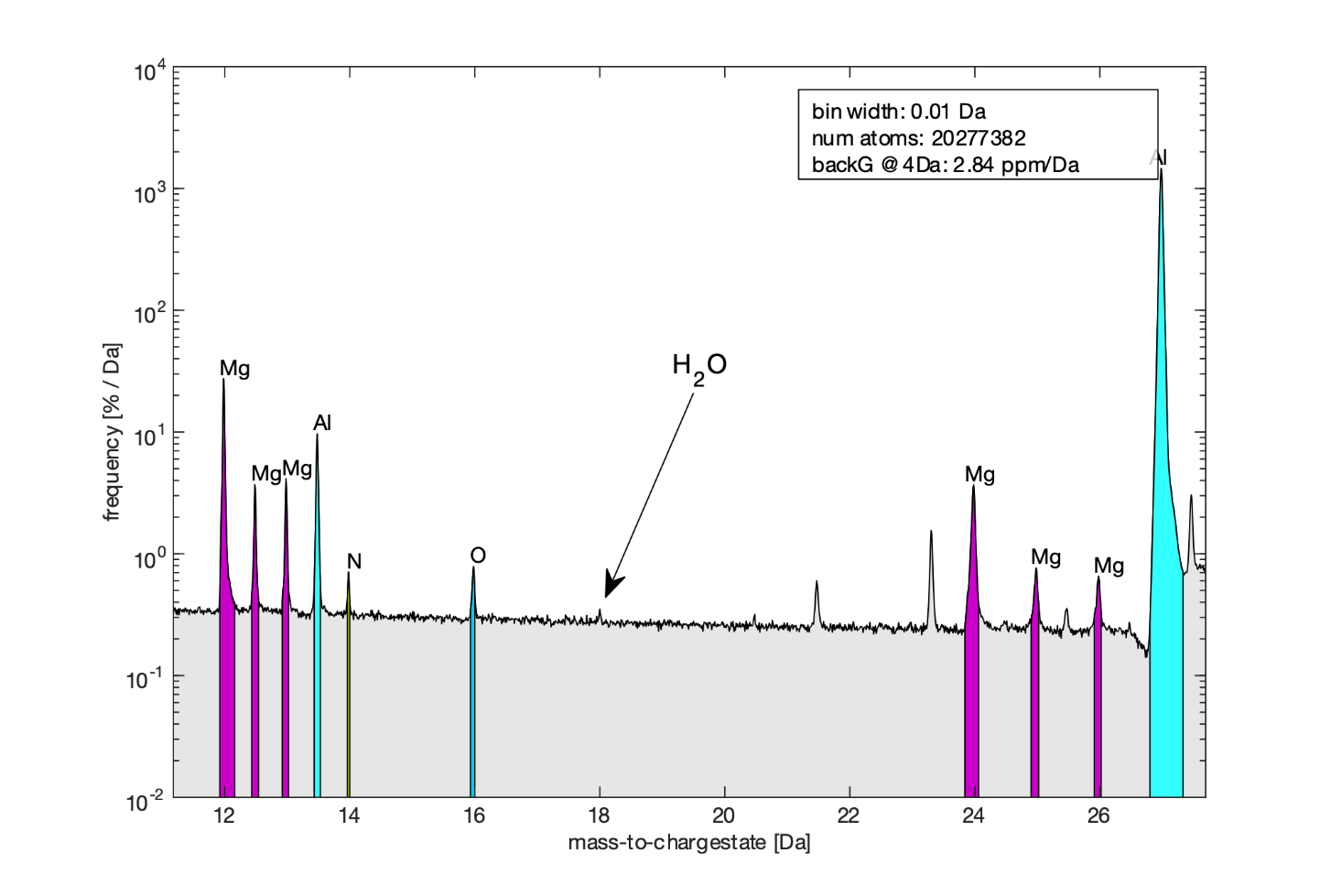


Fig S6, Detail in the mass spectrum near 18 Da (^1^H_2_ ^16^O) shows there are negligible water peaks in the mass spectrum
