# Supplementary material for: A versatile cryo-transfer system, connecting cryogenic focused ion beam sample preparation to atom probe microscopy
Source: PLoS One. 2021 Jan 19;16(1):e0245555. doi: 10.1371/journal.pone.0245555 (PMC7815152; doi:10.1371/journal.pone.0245555)
Supplement: Attachment — Submitted filename: Reviewer Notes.docx [file pone.0245555.s005.docx]

Overall Statement

This study presents results of an in-house design of a unique cryogenic transfer system, primarily connecting a Ga-FIB/SEM to an atom probe. The work also showcases the elimination of Ga segregation to grain boundaries, commonly observed in room temperature ion beam milling and specimen transfer. Although the work overall is very promising and relevant to the scientific community, I do have some concerns related to the content. My primary concerns are: 1) there are some results that I believe need further analysis and clarification before they are ready for publication, 2) the overall presentation of the work is confusing and requires clarification, specifically in the area of naming conventions and overall workflow, and 3) the conclusions seem rather broad and do not directly reflect the findings in the paper.

Detailed Comments

1. The acronym TEM is not introduced in the document. This could be introduced in the 1^st^ paragraph of the introduction.
2. 2^nd^ paragraph of the introduction it sounds as though MPIE has a commercial interest in the Ferrovac system: “In collaboration with CAMECA and the Max Planck Institute for Iron Research in Dusseldorf, Ferrovac offers a large…”. It might be better to say that in collaboration with these companies/institutes that “Ferrovac has developed a …”. (Unless of course MPIE has a commercial interest that I am not aware of…?)
3. The last sentence of the 2^nd^ paragraph in the introduction is a bit vague: “Pacific Northwestern National Laboratory of the US Department of Energy that offers even more possibilities”. Please be more specific about the further possibilities offered by this system.
4. In the sentence ending “through a cryogenic shuttle device designed by the authors, which is introduced below”, remove “which is introduced below” as the first object introduced is the transfer arm, which may then be mistaken for the shuttle device.
5. In the sentence “A general overview of the system including relevant temperatures and pressures at the various steps of sample preparation …”, please add ‘cryogenic/vacuum transfer’ before ‘system’.
6. It is unclear how you would actually plunge freeze the sample prior to transfer to the FIB. Would this be accomplished in some kind of controlled environment?
7. Fig. 1 is very difficult to comprehend and would benefit from rearranging the information. This image is critical to the reader’s capacity to understand the overall interconnectedness of the system and the overall workflow. Care should be taken to make sure it is as clear as possible.
   a. Fig. 1a should be removed and this information incorporated into a single workflow diagram. (Note that temperatures are already incorporated into Figures b,c, and d resulting in double-up of information)
   b. I would suggest placing the FIB/SEM at the top of the image, with operating properties beside it. Separate the transfer arm from the FIB/SEM and place this below the FIB/SEM with the transfer shuttle beside it providing appropriate information for both. Place the LEAP at the bottom (‘CAMECA’ is not really necessary and just takes up space) with appropriate information. Join each component with arrows (as in current Fig. 1a, however make the arrows long enough that the required information can be provided adjacent to them. It would also be clearer if you separated the cryo LL from the LEAP for the purposes of clearly articulating the workflow and isolating the newly designed components from existing infrastructure.
   c. Where you have written -196 dC - RT, please change this to -196 dC to RT, otherwise it looks like an equation
   d. Where and how does plunge freezing fit into this workflow?
8. In the sentence “After FIB milling and/or SEM characterization, the sample is moved out of the FIB/SEM using the same, thermally-insulated, but not actively-cooled transfer arm, in which the sample is handed off to another, actively-cooled transfer arm, with a standard KF16 vacuum flange (Fig 1C)”, the name transfer arm is used to describe two distinct parts of the system. Please be consistent with notation and refer to each component using a distinct naming convention. Alternatively, if you mean that the ‘sample is handed off to another, actively-cooled transfer arm that delivers the sample to the transfer shuttle’ then please be more specific in the detail.
9. In reference to point 8, a number of other items have different names in the text and in the image, please check through the text carefully and be consistent with all naming conventions throughout. The constant changing of names and similarity of names made following the thread of the article extremely difficult.
10. Assuming that the cryo electropolishing station is the station used for initial plunge freezing (discussed in the main text), the image of the cryo electropolishing unit should be included in Figure 1.
11. Fig 2:
    a. Add annotations to a)
    b. Change port names to TS1 and TS2 to represent transfer shuttle 1 and 2. APT 1 and 2 suggest that it will be connected to the atom probe directly.
    c. A better name could be chosen for the cryo-shuttle, at the moment it is too similar to transfer shuttle, which also operates at cryo temperatures.
12. It is not clear how the transfer arm enables the analysis of multiple samples, as indicated in the following sentence “the transfer arm reduces the risk of chamber contamination and enables efficient characterization of multiple samples.” It appears to me that only one sample can be transferred at any one time.
13. “This transfer arm can be used to plunge freeze liquid containing samples prior to characterization (see supplementary materials) and interfaces with the existing Zeiss 80 mm Airlock without further modification”. It is unclear to me how the transfer arm can be used to plunge freeze liquid containing samples without exposure to air. If air exposure is necessary in the process, then ice will build-up on the sample making the method redundant. Please explain further the initial experimental transfer step of samples into the transfer arm under a controlled environment.
14. “This pumping operation takes ca. 40s until a vacuum sufficient for transfer is established.” This is longer than the 30s, stated in the supplementary section, as the maximum time in order not to exceed the crystallization temperature of ice.
15. The supplementary section states the crystallization temperature of water ice to be -160 dC. Please provide a reference, as my understanding was that cryo EM scientists aim to keep the temperature below -150 dC, and that this is sufficient to maintain vitreous ice. Perhaps pressures should be considered in the statement of transition temperatures?
16. 2.2 Atom probe transfer shuttle. This shuttle can be attached to multiple instruments, perhaps a more generic name would be more appropriate.
17. Section 2.2: “So long the reservoir does not run dry, the sample is near liquid nitrogen temperature in the transfer shuttle.” The supplementary information indicates that the minimum temperature of the sample is approx. -170 dC, which is not so near liquid nitrogen temperature. Please add the specific temperature to the text.
18. “However, moving samples from the FIB/SEM with ultimate pressures not below 10-7 mbar, this is not of any concern.” This sentence is confusing, please reword.
19. “enabling temperatures of -160 °C during transfer.” This sentence is now in contrast to the statement, “So long the reservoir does not run dry, the sample is near liquid nitrogen temperature in the transfer shuttle.”.
20. The choice of colour for the tip in Fig 3b makes it difficult to see the tip against a similar background colour. Please change.
21. Fig. 3, consider indicating the location of the sample with an arrow and label for those not familiar with the appearance of APT samples.
22. In section 2.4 you refer to the existing atom probe transfer arm as “LEAP transfer arm”, this becomes particularly confusing now given that the transfer shuttle is often referred to as “LEAP transfer arm”. Please use distinct names.
23. “capable of in-situ heating and cryogenic cooling, and its electrical connections”, ‘and its electrical connections’ doesn’t make sense in this sentence. Please clarify.
24. “can be transferred to a pre-cooled, custom LEAP puck”, it would be better here if you said “can be screwed into a pre-cooled…” to remind readers that the sample is not transferred on a puck but via a double-nipple.
25. “During this hand-over, the LEAP puck is held in place by the actively-cooled cryogenic stage, to make the alignment of the double nipple and the puck easier.” It is not clear from this sentence what is making the alignment of the double nipple and puck easier.
26. “with the long axis of the sample indicated” Please indicate in this sentence that you mean the long axis of the atom probe tip.
27. “This is interesting as H2O is often found in APT data as a contaminant after conventional electrochemical or FIB preparation”. Note that you have Mg_2_O peaks and what looks like a large Mg_2_H_2_O peak between the Ga^2+^ peaks and again MgOH_x_ between 40-50 Da (double check). It is common in Mg containing data not to see H or O peaks, because these elements bond to and evaporate with the Mg in the sample. If you are inclined to continue to mention the lack of H_2_O in the dataset, you must also mention the presence of the Mg-O complex ions in the dataset.
28. Please list the detection rate as a part of the atom probe parameters.
29. Figure 6:
    a. This image is overly compact. Please separate the images or place c and d below a and b.
    b. Make sure the scale bars do not overlap with the images.
    c. Please redo the scale bar for c, I don’t believe this scale bar represents all three axes accurately.
30. Something odd is happening in the video of the desorption map. The edges of the map change significantly throughout the acquisition and poles seem to come and go in relation to the moving of the edges. A few things may cause this: 1) significant vibration of the tip, 2) significant fractures to the tip and 3) slow drifting of the tip. All of which will affect the data. Please address this in the text and discuss implications on the data.
31. I am concerned with the use of Ga^++^ as an indicator for Ga^+^, due to the overlap between Mg_2_O and Ga^++^. This analysis needs to be redone considering this overlap and its implications on Ga concentration. This is particularly of concern given that the peak for the heavier Ga isotope is greater than the peak of the lighter Ga isotope. Furthermore, Ga^++^ does not necessarily always evaporate after FIB preparation, so it could be that these peaks are entirely related to the Mg-O-H peaks.
32. The second paragraph of the conclusion reads more like an introduction than a conclusion. I think that it is necessary to rewrite this paragraph to focus on the specific outcomes of this particular study, such as the observation of Ga free grain boundaries, the rapid transfer times, the shielding of the tip during transfer and the low temperatures maintained during transfer.

Supplementary section

1. The figures are not directly referenced in the text. This is particularly confusing when it is stated that “shown below are the temperature and pressure logs” when the below image is of the experimental set-up. Please be specific in referring to the images in the text.
2. Again further names are given to the shuttle ‘atom probe shuttle’ and ‘LEAP transfer shuttle’, a consistent name is required for the transfer shuttle.
